# A cross-sectional analysis of the impact of lady health worker visits in the prenatal and postnatal period on the uptake of continuum of care interventions and childhood mortality in Pakistan

**DOI:** 10.7189/jogh.15.04158

**Published:** 2025-06-02

**Authors:** Shah Muhammad, Zahid A Memon, Abeer Mian, Yaqub Wasan, Arjumand Rizvi, Imran Ahmed, Sajid Soofi, Simon Cousens, Zulfiqar A Bhutta

**Affiliations:** 1 Centre of Excellence in Women and Child Health, The Aga Khan University, Karachi, Pakistan; 2 Centre for Global Child Health, The Hospital for Sick Children, Toronto, Ontario, Canada; 3 London School of Hygiene and Tropical Medicine London, UK

## Abstract

**Background:**

Community health workers are crucial in bridging the gap between health care facilities and the general population. In Pakistan, the lady health worker (LHW) program was launched in 1994 to enhance access to essential health care services. However, the overall quality of care provided by LHWs and its impact on population-level coverage of key maternal, newborn, and child health (MNCH) interventions and mortality remain insufficiently understood.

**Methods:**

We conducted a cross-sectional analysis using data from 32 106 households with at least one woman of reproductive age across eight districts in Pakistan. Of these households, 63% were located within LHW catchment areas. We categorised households into three groups: 1) no contact with LHWs, 2) at least one contact during either pregnancy or post-delivery, and 3) at least one contact each during both pregnancy and post-delivery.

**Results:**

We observed a clear gradient in the uptake of pregnancy-related and MNCH interventions across the three groups. For instance, four antenatal care visits were reported as 25.3% in group A, 29.4% in group B, and 36.2% in group C (*P* < 0.001). Similar trend followed for skilled birth attendance; 54.4% group A, 58.7% group B, and 64.4% group C (*P* < 0.001). Measles vaccination coverage was 32.3% in group A, 35.2% in group B, and 49.7% in group C (*P* < 0.001). However, there was no evidence of significant differences in neonatal (*P* = 0.862), postnatal (*P* = 0.121), or child mortality (*P* = 0.319) across the three groups.

**Conclusions:**

Increased LHW contact enhances MNCH intervention uptake, though other mechanisms may contribute. Effectiveness depends on service quality, referral systems, and systemic barriers. Strengthening training, optimising referrals, and integrating community health initiatives are vital for sustainability. Addressing workforce shortages, gender challenges, and financial constraints is crucial. Future research should examine sociocultural and programmatic factors influencing health care access and outcomes.

**Registration:**

Clinicaltrials.gov NCT04184544.

The Alma-Ata Declaration introduced the concept of community health workers (CHWs) as key agents of primary health care service delivery with the potential to expand coverage of maternal, newborn, and child health (MNCH) interventions [1,2]. CHWs are widely recognised for bridging the gap between health care facilities and communities, especially for individuals facing barriers to accessing facility-based care. While CHWs are cost-effective to hire and train [3], the World Health Organization (WHO) recommends integrating them within existing health care systems rather than considering them a low-cost substitute for health care providers [4]. When managed and integrated effectively, CHW programs have demonstrated improvements in the quality of care at the household level [5]. Consequently, such programs have been widely adopted globally to address gaps in service delivery [6,7]. Many countries have implemented national CHW programs, each with unique approaches, yet consistently demonstrating improvements in coverage of critical maternal and child health interventions [8].

CHWs vary in their roles and compensation structures across different contexts. Some are unpaid volunteers, while others, like Pakistan’s lady health workers (LHWs), are salaried professionals, albeit with modest pay. Pakistan launched the LHW program in 1994 as part of the national program for family planning and primary health care [9]. The program was designed to improve health indicators through increased utilisation of preventive, promotive, and curative services among vulnerable populations [10]. A significant shift occurred in 2010 when administrative powers were devolved to individual provinces through a Constitutional Amendment, leading to variations in program implementation. While some provinces integrated LHW services into broader maternal and child health initiatives, others maintained the program as a standalone entity [9,10].

Despite the LHW program’s expansion and its recognition for improving MNCH indicators during the Millennium Development Goals period, post-devolution challenges have led to concerns regarding its sustained impact. Administrative and bureaucratic hurdles, including low salaries, job insecurity, and lack of career progression, were highlighted through nationwide LHW protests in 2020 [11]. Additionally, variations in program exposure – measured in terms of LHW visit frequency, scope of services, and household coverage – have contributed to discrepancies in its effectiveness [9–13].

A major limitation in prior evaluations of the LHW program is the reliance on macro-level assessments. While evaluations conducted by Oxford Policy Management provide valuable insights at the provincial and systems levels [12], they lack household-level data that capture the direct impact of LHW interventions on MNCH outcomes. Moreover, existing studies have primarily used observational or administrative data, which are often subject to reporting biases and do not allow for robust causal inference [14]. The devolution of administrative control has further exacerbated data fragmentation, limiting cross-provincial comparisons and comprehensive national assessments of the program’s effectiveness [12].

To address these gaps, we utilised household-level data from the Umeed-e-Nau (UeN) project, implemented across eight districts in Pakistan. This project collected detailed information on women’s health care experiences related to their most recent pregnancy and youngest child [14]. Using cross-sectional baseline data, our study aims to investigate the association between LHW home visits and the uptake of pregnancy-related and MNCH continuum of care interventions, as well as mortality outcomes in newborns and children under five. Unlike prior evaluations, our study employs individual-level data to assess program impact more precisely and explores variations in exposure to LHW services. We hypothesised that regular contact with LHWs is associated with improved coverage of key MNCH interventions, including increased antenatal care (ANC) visits, improved skilled birth attendance, higher immunisation rates, and reduced child mortality. By addressing prior methodological shortcomings, our study contributes to a more nuanced understanding of the LHW program’s effectiveness and informs strategies for optimising CHW-based health care delivery in Pakistan.

## METHODS

This analysis is based on data from a cross-sectional baseline survey conducted as part of a larger quasi-experimental study aimed at scaling up the UeN project [14]. We conducted the baseline survey between March–July 2017 and included a total of 32 106 households from eight districts across Pakistan. Each household included at least one woman of reproductive age (WRA) with a child under the age of five. A full description of the methods used in the UeN baseline household survey has been published as part of the UeN protocol paper [14]. In this analysis, we examined the association between LHW visits during pregnancy and immediately after birth (within 48 hours) and women’s uptake of maternal and child health interventions and child mortality in eight rural districts of Pakistan.

### Study setting

We conducted the baseline survey in eight districts of Pakistan, including three districts from Sindh province, two districts from Punjab province, and three districts from Baluchistan province, covering most of the cultural tapestries and geographical topographies of Pakistan. These districts were selected based on district health information system data indicating high maternal, neonatal, and child morbidity and mortality rates, with a particular focus on predominantly rural areas, as the LHW program primarily serves rural populations. However, these districts are not representative of Pakistan as a whole. During the selection process, a key consideration was ensuring that the chosen districts were not concurrently involved in other major public health programs, thereby avoiding any potential overlap that could affect the outcomes of the UeN program. By targeting high-burden districts, the study aimed to evaluate effective intervention models in settings with significant health challenges, thereby optimising the potential for meaningful improvements in health outcomes.

### Sampling strategy

We employed a two-stage stratified cluster sampling strategy to enrol eligible participants in the survey. First, we compiled a comprehensive list of all villages in each district, including estimated household count and population size, along with their coverage status by the LHW program. We then divided these villages into primary sampling units, each corresponding to the size of the LHW catchment area (cluster), comprising approximately 100–150 households. From this complete list (sampling frame), we selected a random sample of 250 clusters for the survey. Upon identifying a cluster, we conducted a line listing to enumerate all eligible households, *i.e.* secondary sampling unit, a household with an eligible woman. To achieve the required sample size, we selected 20 households per cluster using systematic random sampling. We divided the total number of eligible households in the cluster by 20 to determine the sampling interval. A random starting point was generated within this interval, designating the first household to be visited. The data collection team then proceeded systematically through the selected households to conduct interviews and gather survey data. This rigorous approach enhanced the study’s precision and generalizability while maintaining logistical feasibility.

### Data collection

In the baseline survey, we utilised a standardised Demographic and Health Survey questionnaire, covering demographic and household characteristics, details of the most recent childbirth, and care-seeking during pregnancy, delivery, and the postpartum period. It also included questions on the health and health care of the youngest child. We applied the same tool and methodology across both intervention and control districts to ensure uniformity and data reliability. Before data collection, we tested the pilot questionnaire in a similar field setting to assess its validity and reliability. We analysed the pilot data to confirm that the questionnaire effectively captured the intended study outcomes. A previous study had also used a similar approach to assess LHW contacts and household-level outcomes [15].

Data collectors underwent a five-day training program covering questionnaire administration, ethical considerations, and strategies to minimise interviewer bias. This training included three days of classroom instruction and two days of field testing. We conducted mock interviews to ensure accuracy and consistency. We pilot-tested the questionnaire with 20 participants from a similar demographic to assess clarity, validity, and cultural appropriateness, with necessary modifications made based on feedback before final data collection.

To maintain data quality, study field supervisors conducted on-the-spot monitoring visits for 10% of the households using pre-designed checklists. Any inconsistencies identified were addressed through real-time feedback to field teams, enhancing data accuracy and reliability. Given the lower literacy rates in the study population, data were collected verbally in local languages using handheld devices equipped with a customised application developed in Java with SQLite for data storage. Built-in consistency and completeness checks were implemented to minimise errors and ensure data quality.

We obtained informed consent from all respondents after explaining the study’s purpose, potential risks and benefits, confidentiality measures, and their right to decline answering any question or withdraw at any stage. The importance of honest and candid responses for shaping potential MNCH interventions was also emphasised. These measures were taken to minimise response biases, such as social desirability bias. Additionally, to reduce recall bias, we collected MNCH data specifically for the most recent pregnancy and child.

### Ethical approval

Aga Khan University Institutional Ethics Review Committee and Pakistan National Bioethics Committee approved the study (reference numbers 4468-Ped-ERC-16 and 4-87/17/NBC-224/NBC). Additionally, we registered the study with Clinical Trial (NCT04184544). Moreover, all study respondents provided informed consent.

### Data analysis

When assessing the coverage of care, we assessed the ANC coverage in terms of visits made by women to a skilled provider (a physician, lady health visitor, or nurse) at a health care facility. We did not consider visits by LHWs during the prenatal period as antenatal visits. We categorised the exposure variable into three levels based on LHW functionality status during prenatal and postnatal visits conducted by an LHW, defined as follows:1) not functional (LHW did not visit during prenatal or postnatal period), 2) partially functional (LHW visited either during the prenatal or postnatal period), and 3) fully functional (LHW visited during both prenatal and postnatal periods). For each group of women, we calculated and compared coverage rates for the following interventions along the maternal and child health continuum of care: 1) ANC four facility visits, 2) skilled birth attendance, 3) facility-based delivery, 4) early initiation of breastfeeding, 5) postnatal checkup within two days (for mother and child), 6) Bacillus Calmette-Guérin (BCG) vaccine, measles vaccine, full immunisation status (age-appropriate), 7) care-seeking for diarrhoea, use of oral rehydration solution, and 8) care-seeking for acute respiratory infection.

We compared the groups using univariate methods (survey-adjusted χ^2^ test). We used logistic regression to determine both crude and adjusted mortality rates and *P*-values to compare LHW functionality groups. We defined neonatal mortality as newborn death within the first 28 days of life, post-neonatal mortality as death occurring from the first month to 59 months of life, and overall under-five mortality as death from birth to 59 months of life. We adjusted these rates for household socio-economic status, distance from the household to the health facility, women's education, and parity. We adjusted all analyses to account for survey design, including. clustering and sampling weight.

## RESULTS

Overall, 19 094 (60.5%) of the study population resided in LHW catchment areas, while the remaining 12 431 (39.5%) were in areas uncovered by LHW. In LHW-covered areas, the majority of WRA (66.6%) were visited by LHW during either the antenatal or postnatal period. Around one-fifth (18.6%) were not visited during either period, while around 15% of the pregnant women were visited during both periods. We observed notable differences between provinces in the distribution of the study population concerning LHW availability and contact. Baluchistan had the lowest percentage of households that reported visits from the LHW during either the prenatal or postnatal period (43.2%), whereas in Punjab and Sindh, 72.4% and 60.5% of households, respectively, received either prenatal or postnatal care. The proportion of WRA who received both prenatal and postnatal visits was similar in Punjab and Sindh, with about 15.0% of households receiving LHW contact during those periods. Conversely, a small proportion (5.6%) reported having both contacts in Baluchistan (**Table 1**).

**Table 1:** Demographic characteristics of survey population by LHW functionality group*

Characteristics	Overall (n = 19 094)	No LHW visit during prenatal or postnatal period (n = 5813)	LHW visited either for the prenatal or postnatal period (n = 10 882)	LHW prenatal and postnatal visit (n = 2399)
Province				
*Baluchistan*	5295 (7.7)	2972 (56.8)	2015 (37.6)	308 (5.6)
*Punjab*	5625 (54.2)	771 (12.3)	3993 (72.4)	861 (15.3)
*Sindh*	8174 (38.0)	2070 (24.0)	4874 (60.5)	1230 (15.5)
Location				
*Urban*	4979 (17.1)	1782 (24.0)	2654 (62.8)	543 (13.1)
*Rural*	14 115 (82.9)	4031 (17.1)	8228 (67.6)	1856 (15.3)
Mother education level				
*No education*	13 142 (73.8)	4224 (19.8)	7311 (65.7)	1607 (14.5)
*Primary (1–5 years)*	2383 (11.6)	626 (15.6)	1429 (68.3)	328 (16.1)
*Above primary (>5 years)*	3569 (14.6)	963 (16.6)	2142 (68.4)	464 (15.0)
Wealth index (quintiles)				
*Poorest*	3591 (20.3)	1212 (20.1)	2129 (72.3)	250 (7.6)
*Poor*	3747 (20.0)	1203 (19.6)	2161 (68.3)	383 (12.1)
*Middle*	3818 (19.7)	127 (18.4)	2190 (65.8)	501 (15.8)
*Rich*	3891 (20.0)	1123 (17.4)	2179 (64.9)	589 (17.7)
*Richest*	4047 (20.2)	1148 (17.7)	2223 (62.0)	676 (20.2)

LHW – lady health worker

*Presented as n (%).

### Uptake of key pregnancy and MNCH-related interventions

There was significant evidence of differences in indicators between areas visited by LHWs during either the prenatal or postnatal period, or both, compared to those that were not visited during either period. A clear gradient in the uptake of key pregnancy-related interventions was observed across the continuum of care, with statistically significant differences among the groups.

For example, the proportion of women who had at least four ANC visits was 25.3% in group A, 29.4% in group B, and 36.2% in group C (*P* < 0.001). Similarly, skilled birth attendance was reported at 54.4% in group A, 58.7% in group B, and 64.4% in group C (*P* < 0.001). However, we found no significant difference in the reported practice of early breastfeeding initiation (**Table 2**).

**Table 2:** Differences in coverage of key MNCH interventions by LHW visits within LHW-covered area across the continuum of pregnancy-related care*

Interventions	No LHW visit during prenatal or postnatal period (n = 5813)	LHW visited either for prenatal or postnatal periods (n = 10 882)	LHW visited during both the prenatal and postnatal periods (n = 2399)	*P*-value
At least 4 ANC visits	1410 (25.3)	3109 (29.4)	831 (36.2)	<0.001
Skill birth attendant	3211 (54.4)	6314 (58.7)	1543 (64.4)	<0.001
Facility-based delivery	3072 (53.0)	6150 (57.5)	1502 (62.0)	<0.001
Caesarean section	774 (17.1)	1894 (18.8)	463 (19.9)	0.111
Early initiation of breastfeeding	1190 (19.9)	2111 (18.9)	457 (19.2)	0.547
Post-natal visit for mother within 48 h	823 (14.2)	1693 (15.1)	508 (22.4)	<0.001
Post-natal visit for the child within 48 h	1179 (19.6)	2429 (21.0)	831 (37.0)	<0.001

ANC – antenatal care, LHW – lady health worker

*Presented as n (%).

**Figure 1:**
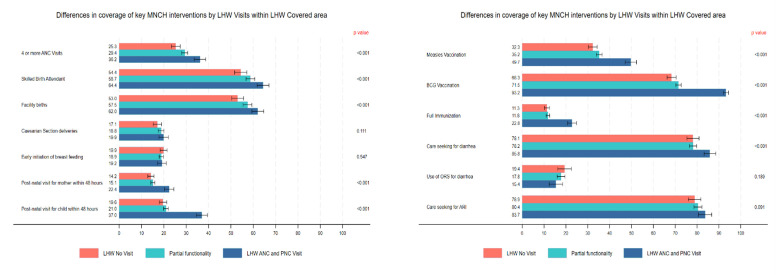
Differences in coverage of key MNCH interventions by LHW Visits within the LHW-covered area across the continuum of pregnancy-related care. CI – confidence interval.

We observed a similar pattern in the utilisation of MNCH interventions. For example, measles vaccination coverage was 32.3% in group A, 35.2% in group B, and 49.7% in group C (*P* < 0.001). Additionally, a significantly larger proportion of children in group C received the BCG vaccination, where LHWs were fully functional or frequently engaged with WRA (68.3% in group A, 71.5% in group B, and 93.2% in group C (*P* < 0.001). However, we found no significant differences in the use of oral rehydration solution for diarrhoea or in care-seeking for acute respiratory infections (**Figure 1**).

### Child mortality

Adjusted and unadjusted multivariate regression analyses showed no statistically significant differences in neonatal (*P* = 0.862), postnatal (*P* = 0.121), or child mortality (*P* = 0.319) among the groups (**Table 3**).

**Table 3:** Differences in mortality by health workers’ visits within LHW-covered area

Items	Unadjusted		Adjusted*	
**Rate (95% CI)**	***P*-value**	**Rate (95% CI)**	***P*-value**
Neonatal deaths		0.27		0.862
*LHW no visit*	31.8 (25.0–38.5)		32.3 (25.5–39.2)	
*LHW visited either for the prenatal or postnatal period*	30.2 (25.4–34.9)		30.6 (25.9–35.3)	
*LHW visited both the prenatal and postnatal periods*	30.5 (21.8–39.2)		29.5 (21.1–38.0)	
Postnatal deaths		0.133		0.121
*LHW no visit*	32.0 (23.8–40.2)		31.7 (23.3–40.1)	
*LHW visited either for the prenatal or postnatal period*	37.4 (32.3–42.5)		37.0 (32.0–42.1)	
*LHW visited both the prenatal and postnatal periods*	28.5 (19.2–37.9)		28.3 (19.4–37.2)	
Under-five mortalities		0.421		0.319
*LHW no visit*	63.8 (54.0–73.5)		64.1 (54.3–73.8)	
*LHW visited either for the prenatal or postnatal period*	67.6 (60.9-74.3)		67.6 (61.0–74.2)	
*LHW visited both the prenatal and postnatal periods*	59.1 (47.1–71.0)		57.9 (46.8–69.1)	

CI – confidence interval, LHW – lady health worker

*Adjusted for wealth terciles, distance to nearest facility, mother’s education and number of children.

## DISCUSSION

Our assessment of coverage data highlights significant differences in maternal and child health intervention utilisation based on the frequency of LHW visits. Women with more frequent LHW contact demonstrated better health care utilisation during pregnancy, post-delivery, and for child health. These findings align with existing literature. For instance, skilled birth attendance rates increased progressively from 54.0% in group A (no LHW contact) to 58.7% in group B (at least one contact) and 64.4% in group C (both pregnancy and postnatal contact) (*P* < 0.001). A quasi-experimental study in rural Kenya similarly found that exposure to CHWs significantly improved maternal and newborn care-seeking behaviours [16].

Participants in group C also showed better child health indicators, particularly in immunisation (*P* < 0.001), diarrhoea-related care-seeking (*P* < 0.001), and postnatal care-seeking (*P* < 0.001). This underscores LHWs’ critical role in facilitating health care access. One explanation is that LHWs actively support routine immunisation and liaise with vaccinators to ensure child vaccination, particularly in areas with limited health care personnel. Studies in Kenya and India confirm that CHW exposure nearly doubles the likelihood of receiving recommended vaccinations, including the measles vaccine [17,18]. Similarly, in Pakistan, LHWs are recognised as the primary source of child health information at the community level [19]. They provide various services, including immunisation, child illness management, breastfeeding counselling, and growth monitoring [12,19]. Furthermore, district-level analyses reveal a strong correlation between LHW density and vaccination coverage [20,21].

A global systematic review of randomised controlled trials in low- and lower-middle-income countries underscores CHWs’ contributions to improved maternal and child health outcomes. These include increased vaccination rates, higher exclusive breastfeeding prevalence, reduced neonatal and maternal mortality, and improved family planning adoption [22]. Evidence from Southern Africa further highlights CHWs’ role in neonatal care practices, including umbilical cord care, thermoregulation, hand hygiene, and HIV prevention [23,24]. Expanding LHW reach in rural areas is therefore crucial, yet maximising their impact requires formal policy recognition, capacity-building strategies, and integration into the broader health system to ensure sustainability. Beyond LHW engagement, sociocultural factors also shape health care-seeking behaviours. Cultural norms, male decision-making authority, and alternative health care options influence maternal and child health outcomes. Addressing these contextual barriers is necessary to optimise LHW program effectiveness. The debate surrounding CHW compensation further complicates their sustainability. While some argue that financial incentives may undermine intrinsic motivation and strain budgets, others emphasise that an unpaid model contradicts Sustainable Development Goal eight, which promotes economic growth and decent work opportunities [25]. The fourth Oxford Policy Management evaluation raises concerns about program sustainability [12]. While federal funding via the planning commission one initially mitigated financial constraints, policy reversals continue to threaten the LHW program [26]. Additionally, gender inequities hinder LHWs’ professional growth. The predominantly female workforce faces safety concerns, excessive workloads, and low financial compensation, contributing to high turnover and limiting their impact. Societal resistance, gender biases, and inadequate rural infrastructure further restrict their outreach and effectiveness. Addressing these structural and systemic barriers is essential to ensure LHWs can operate safely and efficiently [27].

Health system weaknesses also constrain LHW impact. Poor referral mechanisms and suboptimal facility-based care may counteract LHW efforts to improve maternal and child health. For instance, encouraging mothers to visit health care facilities will have little long-term impact if they encounter poor-quality or disrespectful care. While they may visit once, negative experiences will likely deter them from returning. Strengthening these systemic components is necessary to fully leverage CHW interventions. Global models for CHW remuneration provide valuable insights into sustainable engagement. Countries employ diverse approaches, including public sector salaries (Brazil), volunteer-based models (Ghana), private sector minimum wages (Nigeria), performance-based cooperative incentives (Rwanda), and hybrid public-private salary structures (South Africa) [28]. A structured, hybrid remuneration model may ensure both sustainability and fair compensation, fostering motivation and retention among LHWs.

Despite financial limitations, evidence from Southern Africa and Asia demonstrates the effectiveness of community-based interventions in reducing newborn and maternal mortality by 15–20% through antenatal and postnatal home visits [15,29,30]. India’s Accredited Social Health Activist program further confirms that CHW-led engagement increases ANC and skilled birth attendance [31]. Consistent with national guidelines recommending four ANC visits [32], we found significantly higher adherence among group C participants (36.2%) than group B (29.4%) and group A (25.3%) (*P* < 0.001). These findings echo a randomised controlled trial in Afghanistan, where CHW deployment increased ANC visit uptake by 10.5% [33].

The Oxford Policy Management evaluation reflects measurable improvements in LHW performance, with the LHW performance score rising from 42 to 52. Their counselling and doorstep services have enhanced antenatal consultations, institutional deliveries, and complication reporting during pregnancy and postpartum [26]. However, despite these gains, LHWs’ overall impact declined post-devolution in 2011, with no observed improvement in child immunisation rates [20]. We found no significant association between LHW coverage and mortality, despite prior evidence linking CHW interventions to mortality reduction. A 2011 cluster-randomised trial in Sindh demonstrated that LHW-led community group sessions significantly reduced stillbirths and neonatal mortality [15]. Meta-analyses of eight studies further estimate a 24% reduction in neonatal mortality due to CHW-provided home-based newborn care [34]. However, a 2017 cross-sectional study on the UeN project found no significant difference in neonatal mortality between areas with and without adequate LHW coverage, although it did associate LHW presence with lower child mortality risk [35]. These findings highlight the need for further research to refine program implementation and maximise impact. Structural limitations within the LHW programme may explain these mixed results. The Oxford Policy Management evaluation identifies weak strategic oversight, including the absence of mid-term evaluations, inactive high-level committees, and frequent leadership turnover, leading to inconsistent program direction [26]. While training initiatives and pilot studies have yielded positive results, mechanisms for expanding LHW services remain underutilised. The program review committee, responsible for approving LHW involvement in new service areas, has remained largely inactive, and clinical priority guidelines have not been developed. These gaps undermine LHW effectiveness in reducing neonatal mortality [26].

Moreover, LHWs have limited access to skilled training for emergency obstetric care. Their curriculum lacks critical components such as sanitation, waste disposal, and emergency health response [36]. Additionally, inadequate referral pathways and inconsistencies in facility-based care quality further constrain their impact on maternal and child health. Poor knowledge dissemination regarding conditions like pre-eclampsia suggests that LHW interactions may not be sufficiently impactful, emphasising the need for improved training and communication strategies [37]. LHWs also face significant logistical barriers to attending home births, including safety risks, lack of travel support, and financial disincentives. These constraints prevent them from providing timely maternal and newborn care, reinforcing the need for targeted policy interventions [27].

We offer evidence-based insights into LHWs’ role in improving coverage of maternal and child health interventions at the community level. However, declining LHW contact rates present a challenge, driven by workforce reductions, particularly in Balochistan, where LHW numbers have declined by 21.0% [38,39]. This workforce shortage is reflected in our findings, with Balochistan reporting the lowest LHW contact rates compared to Punjab and Sindh. Sociocultural barriers further hinder service delivery, with mobility restrictions and gender-based exclusions limiting outreach [40].

This study is among the first to assess LHW impact using robust and relevant variables. However, it has limitations, including reliance on self-reported recall, lack of qualitative insights into LHWs’ and women’s grassroots challenges, and exclusion of male perspectives, which play a crucial role in health care decision-making. Future research should explore these dimensions to inform more comprehensive community health strategies.

## CONCLUSIONS

Our findings underscore the crucial role of LHWs in improving MNCH outcomes by increasing ANC utilisation, facility-based deliveries, postnatal care uptake, immunisation coverage, and skilled care-seeking behaviours. However, the effectiveness of these interventions varies based on the frequency of LHW engagement, highlighting the need for sustained and structured LHW involvement. While LHWs significantly enhance health care access, our study did not establish a clear association between LHW coverage and child mortality reductions, emphasising the need for further investigation into service quality, referral pathways, and health system integration.

Ensuring that LHW-led interventions translate into tangible health improvements requires addressing systemic and structural barriers. Strengthening LHW training through standardised, competency-based curricula – including emergency obstetric care and newborn health management – can enhance service delivery. Additionally, formalising referral pathways and reinforcing linkages with primary health care facilities will improve continuity of care. Robust monitoring and evaluation mechanisms, such as digital tracking of services and community-based feedback systems, are essential to maintaining accountability and quality standards.

Beyond technical improvements, addressing sociocultural and structural challenges is critical. Gender-based restrictions, safety concerns, workforce shortages, and financial constraints limit LHW effectiveness, particularly in rural and underserved areas. Policy reforms must prioritise fair remuneration models, professional development opportunities, and gender-sensitive strategies to ensure both sustainability and equity in LHW programs.

The declining prioritisation of maternal and child health at the national level, coupled with workforce reductions – particularly in Balochistan – poses significant threats to program sustainability. Policymakers must commit to long-term investment in LHW programs through resource allocation at the community, facility, and district levels. Expanding LHW coverage, improving supervision structures, and integrating community health initiatives within broader health system reforms can maximize impact and ensure equitable health care access for all.

Future research should explore the qualitative dimensions of LHW service delivery, including women’s and LHWs’ lived experiences, male decision-making influences, and sociocultural determinants of health care-seeking behaviour. A comprehensive, context-specific approach to LHW engagement – one that combines evidence-based interventions with structural reforms – will be essential in bridging the gap between health care access and improved maternal and child health outcomes.
